# MRI-detected extramural venous invasion of rectal cancer: Multimodality performance and implications at baseline imaging and after neoadjuvant therapy

**DOI:** 10.1186/s13244-021-01023-4

**Published:** 2021-08-09

**Authors:** Akitoshi Inoue, Shannon P. Sheedy, Jay P. Heiken, Payam Mohammadinejad, Rondell P. Graham, Hee Eun Lee, Scott R. Kelley, Stephanie L. Hansel, David H. Bruining, Jeff L. Fidler, Joel G. Fletcher

**Affiliations:** 1 Department of Radiology, Mayo Clinic, Rochester, 200 First Street SW, Rochester, MN 55905 USA; 2 Department of Laboratory Medicine and Pathology, Mayo Clinic, Rochester, 200 First Street SW, Rochester, MN 55905 USA; 3 Division of Colon and Rectal Surgery, Mayo Clinic, Rochester, 200 First Street SW, Rochester, MN 55905 USA; 4 Division of Gastroenterology and Hepatology, Mayo Clinic, Rochester, 200 First Street SW, Rochester, MN 55905 USA

**Keywords:** Extramural venous invasion, Rectal Neoplasms, Prognosis, Disease-free survival, Magnetic resonance imaging

## Abstract

MRI is routinely used for rectal cancer staging to evaluate tumor extent and to inform decision-making regarding surgical planning and the need for neoadjuvant and adjuvant therapy. Extramural venous invasion (EMVI), which is intravenous tumor extension beyond the rectal wall on histopathology, is a predictor for worse prognosis. T2-weighted images (T2WI) demonstrate EMVI as a nodular-, bead-, or worm-shaped structure of intermediate T2 signal with irregular margins that arises from the primary tumor. Correlative diffusion-weighted images demonstrate intermediate to high signal corresponding to EMVI, and contrast enhanced T1-weighted images demonstrate tumor signal intensity in or around vessels. Diffusion-weighted and post contrast images may increase diagnostic performance but decrease inter-observer agreement. CT may also demonstrate obvious EMVI and is potentially useful in patients with a contraindication for MRI. This article aims to review the spectrum of imaging findings of EMVI of rectal cancer on MRI and CT, to summarize the diagnostic accuracy and inter-observer agreement of imaging modalities for its presence, to review other rectal neoplasms that may cause EMVI, and to discuss the clinical significance and role of MRI-detected EMVI in staging and restaging clinical scenarios.

## Key points

- T2WI demonstrates EMVI as an expanded, irregular vessel with intermediate tumor-signal intensity.
- CT can sometimes depict EMVI as a heterogeneously enhancing, serpentine cord-like structure
- Inter-observer agreement in assessing the presence or absence of EMVI is variable.
- EMVI is a predictor of poor prognosis and indicates biologic aggressiveness.
- EMVI may be seen in association with rectal  tumors other than adenocarcinoma.

## Background

Colorectal cancer is the third most common neoplasm in adults, with approximately 43,000 patients diagnosed with rectal cancer in the United States in 2020 [1]. MRI provides detailed imaging of rectal cancers and relevant pelvic structures and is routinely used to evaluate tumor extension beyond the muscularis propria of the rectal wall as well as involvement of the mesorectal fascia [2–4]. Rectal cancer MRI is routinely used to inform therapeutic decisions, e.g., the use and type of neoadjuvant therapy, oncologic surgical planning, and preoperative assessment after neoadjuvant therapy [5].

Direct tumor invasion into the extramural veins on histopathology, known as extramural venous invasion (EMVI), has been recognized as an indicator of poor prognosis [6, 7]. Brown et al. found that MRI-detected EMVI correlated well with histopathological EMVI [8]. MRI-detected EMVI is now widely accepted as an independent poor prognostic factor for disease-free survival. Consequently, MRI-detected EMVI is an important consideration in therapeutic decision-making similar to histopathological EMVI [9]. Despite the fact that diagnosis can be challenging, radiologists should evaluate rectal cancers for EMVI at pelvic MR as it is an important imaging biomarker for nodal or distant metastasis, unfavorable prognosis, and need for neoadjuvant therapy [10].

This article reviews the imaging findings of EMVI on MRI and CT, summarizes the diagnostic accuracy and inter-observer agreement for detection of EMVI, and reviews the clinical significance and role of MRI-detected EMVI in staging and restaging scenarios with an aim to disseminate a common understanding of EMVI and its varied appearances.

### Venous anatomy of the rectum

**Fig. 1:**
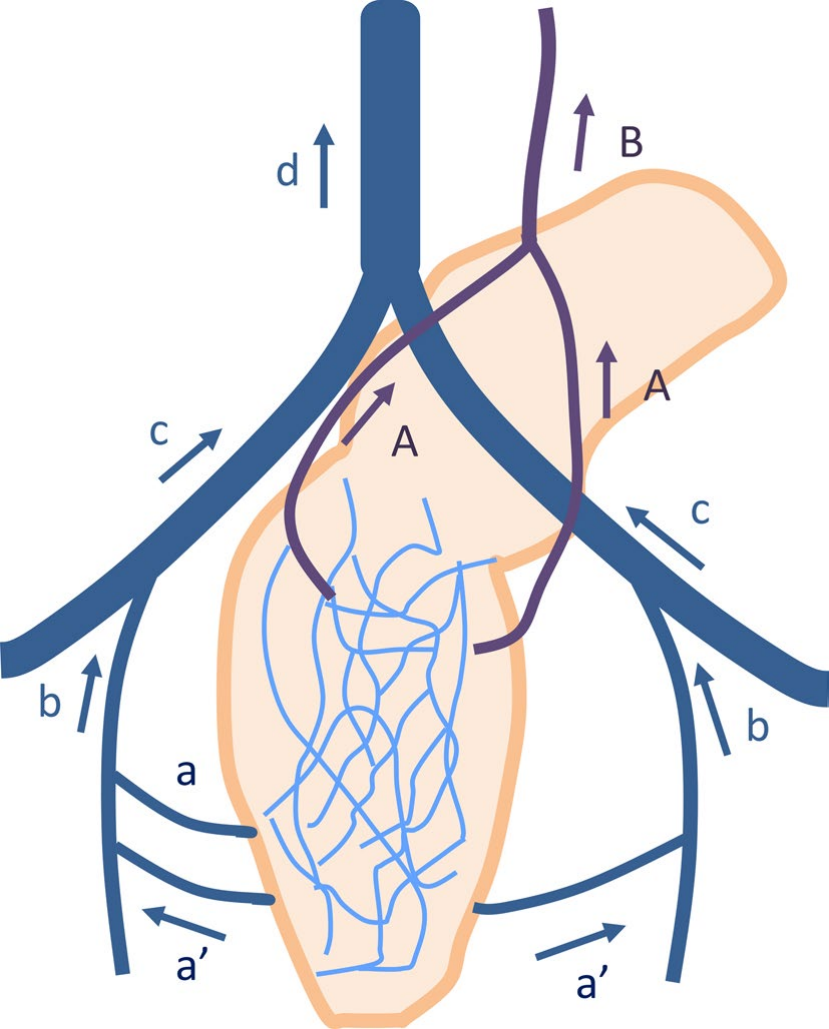
Venous anatomy of the rectum. The upper 2/3 of the rectum drains via the superior rectal vein (**A** SRV) into the inferior mesenteric vein (**B** IMV). The lower 1/3 of the rectum drains via the middle (**a** MRV) and inferior rectal vein (**a’** IRV) into the internal iliac vein (**b** IIV), common iliac vein (**c** CIV), and inferior vena cava (**d** IVC)

Understanding the rectal venous plexus anatomy can be helpful when aiming to accurately identify EMVI on MRI. The venous drainage of the anorectum occurs via two interconnected plexuses, the inner submucosal plexus of the anorectum (the internal rectal plexus) and the outer muscularis plexus (the external rectal plexus). Venous drainage of the upper 2/3 of the rectum is via the superior rectal/hemorrhoidal veins, which drain into the inferior mesenteric vein [11]. Venous drainage of the lower 1/3 of the rectum is via the middle and inferior rectal veins, which drain into the internal iliac veins, common iliac veins, and inferior vena cava (Fig. 1). The middle rectal vein is found in 32.6% of individuals, and is unilateral in the majority [12]. Thus, venous return from the rectum enters both the portal and systemic circulation.

### EMVI in histopathology

**Fig. 2:**
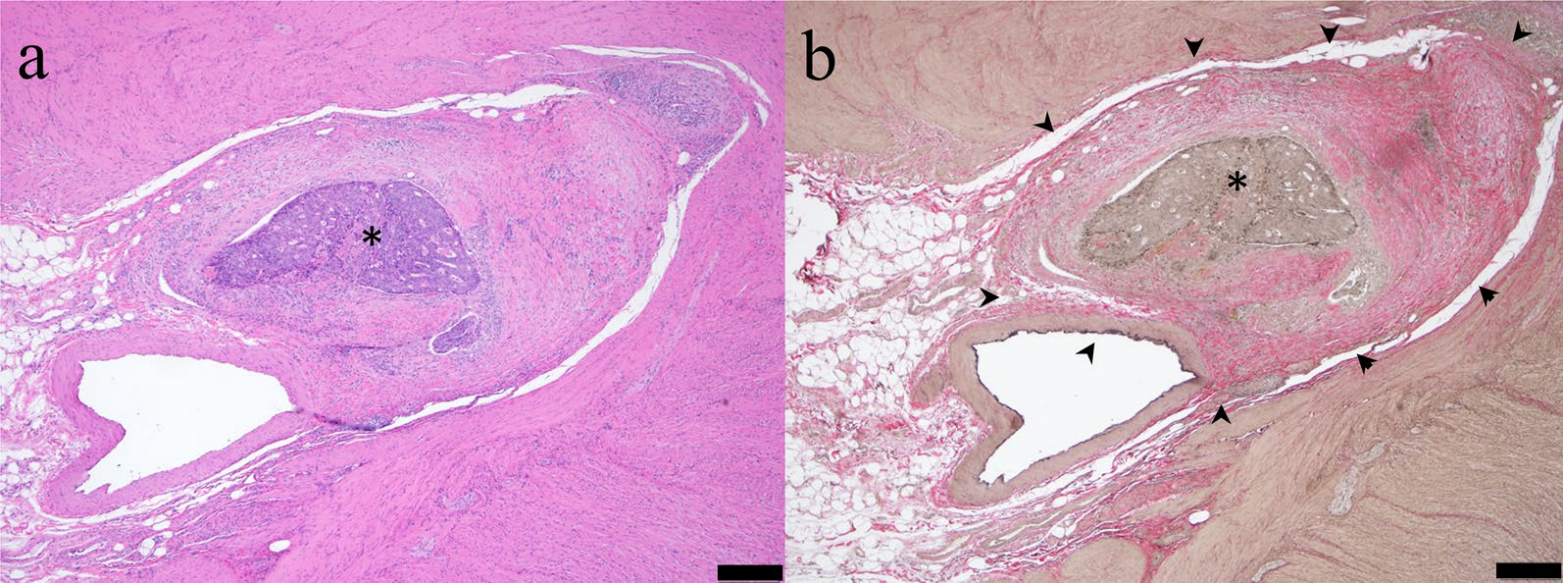
Extramural venous invasion on histopathology. **a** The tumor cells (asterisk) are surrounded by a vessel on hematoxylin–eosin stain. **b** Elastin stain is helpful to depict extramural venous invasion (asterisk) by highlighting elastin fiber (arrowheads) around tumor cells. The scale bar is 500 µm

EMVI is defined histopathologically as the presence of tumor cells within blood vessels located beyond the muscularis propria of the rectal wall (Fig. 2a); however, desmoplastic reaction and endothelial destruction induced by tumor invasion destroy the vessel wall and can preclude identification of venous anatomy, making EMVI challenging to detect even at histopathology [13]. An elastin stain highlights elastic fibers present in the adventitia of veins (but not lymphatics), and improves the identification of EMVI compared to routine hematoxylin–eosin stain alone (Fig. 2b) [14, 15]. The detection of venous invasion is dependent on the number of examined tissue blocks or slides [16]; therefore, imaging detection of EMVI can guide the pathologist in selecting the location and number of tissue samples for slides.

Invasion of tumor into blood vessels is considered to be an initial step in hematogenous metastasis [17], and histologic venous invasion of colorectal carcinoma was identified as a risk factor for metastatic disease in the 1930s [6]. Venous invasion is categorized as intramural or extramural based on whether the location is within or beyond the bowel wall. Histopathologically-detected EMVI portends a worse 5-year survival rate and an increased risk of hepatic metastasis compared to intramural venous invasion, which demonstrates a worse prognosis than no venous invasion [7, 16]. The presence of EMVI on resection specimens following neoadjuvant therapy is associated with a significantly worse prognosis in patients with rectal cancer [18, 19]. If a patient did not receive neoadjuvant therapy prior to surgery, and the surgical histopathology shows high risk features, including EMVI, chemotherapy is often recommended following surgery.

### EMVI in magnetic resonance imaging

The MERCURY study evaluated high-resolution rectal MRI with an in-plane resolution of 0.6 × 0.6 mm and reported that MRI could predict surgical margin status [20] and extramural depth equivalent to that on pathology with a difference less than 0.5 mm [3]. Using this same optimized scan protocol with high-resolution T2WI, Brown and colleagues also first described EMVI in the setting of rectal cancer and noted its presence to be an important prognostic finding, similar to EMVI on histopathology [8]. Rectal MRI is considered capable of depicting EMVI greater than 3 mm despite the spatial resolution of MRI being limited compared with histopathology [8].

**Fig. 3:**
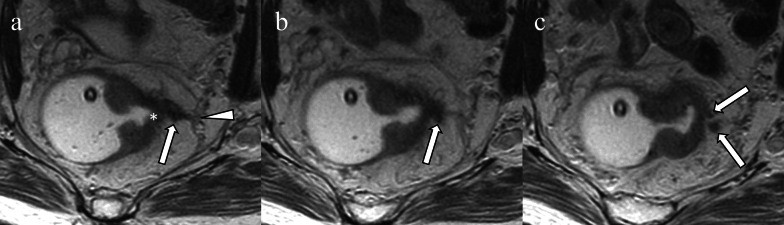
Extramural venous invasion on T2-weighted image. A nodular-shaped structure (**a** arrow) arising from the ulcer crater and leading edge of the primary lesion (**a** asterisk) invades the mesorectal fascia (**a** arrowhead). The structure also  extends cranially in the mesorectal fascia (**b** arrow) and is branched (**c** arrows)

EMVI at MRI is usually associated with primary tumor extending beyond the muscularis propria and into the mesorectal fat. The leading edge of the tumor with such extramural extension and associated EMVI is typically nodular or sawtooth  rather than smooth. The infiltrated vessel is contiguous with the primary tumor, may be expanded, and has lost the normal black signal flow void, which is replaced with intermediate signal T2 tumor intensity. The vessel often has irregular margins and may appear beaded or nodular on T2 and post contrast imaging (Fig. 3) [5, 21].

**Fig. 4:**
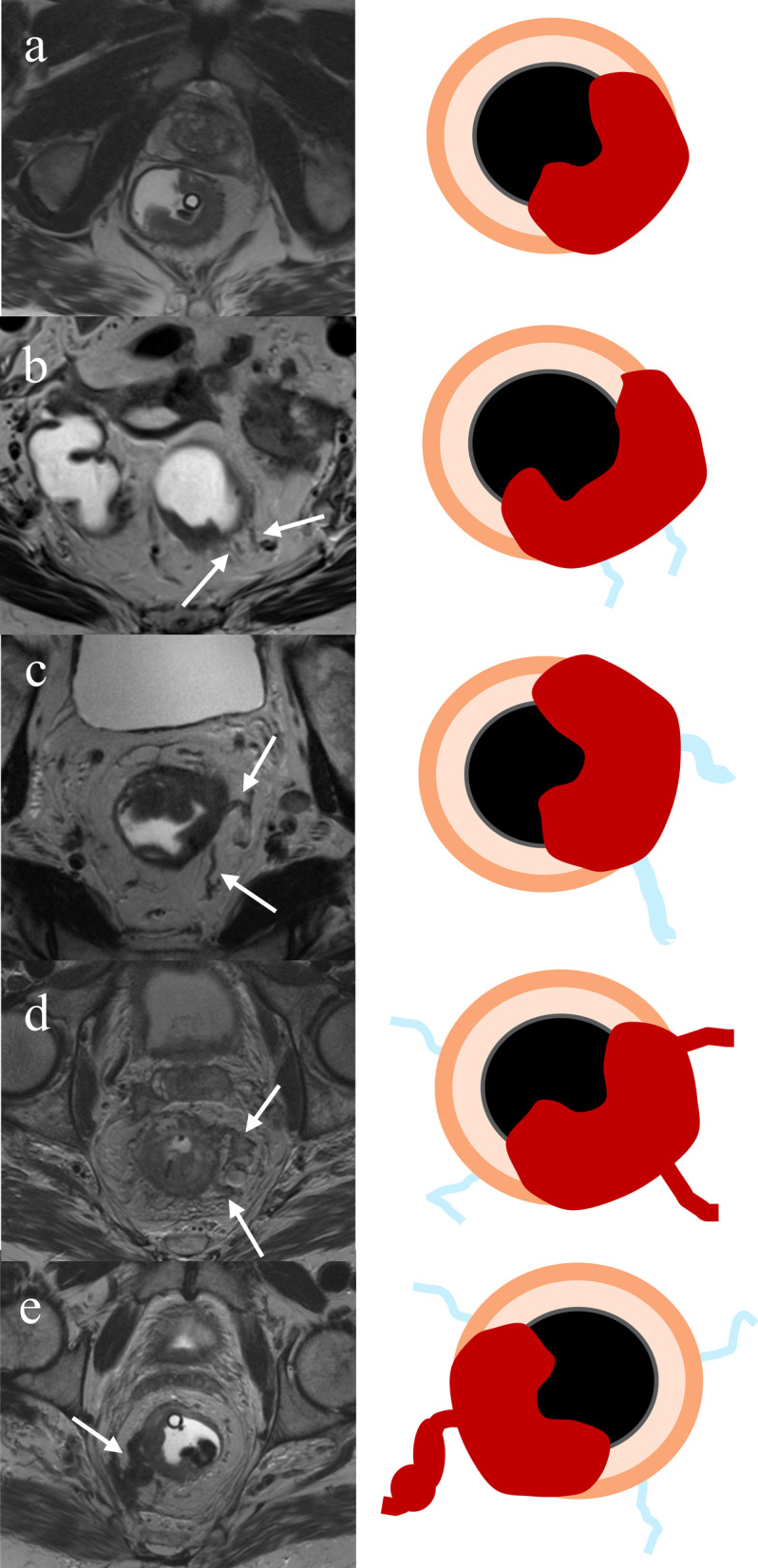
Extramural venous invasion scoring system based on T2-weighted images. **a** No vessel exists adjacent to the primary tumor (score 0). **b** Normal diameter vessel adjacent to the primary tumor demonstrates no tumor signal intensity (score 1). **c** Slightly expanded vessel without abnormal signal intensity (score 2). **d** Expanded vessel including obvious tumor signal intensity (score 3). **e** Expanded vessel with irregular or nodular contour containing tumor signal intensity (score 4). Adapted from Smith NJ, et al. Br J Surg 2008 [22]

To help in assessing EMVI, Smith et al. developed a 5-point scoring system on T2WI based on the aforementioned MR imaging features [22]. This scoring system stratifies a broad spectrum of findings into five categories and has been used in several research studies [22–32]. Score 0 is defined as tumor extension through the muscle layer, without nodularity, and lacking vessels adjacent to the area of tumor penetration. Score 1 is defined as minimal extramural stranding and nodular extension, but not in the vicinity of any vascular structure. Score 2 is defined as stranding in the perirectal fat in the vicinity of normal diameter extramural vessels. Score 3 is defined by intermediate T2 signal intensity within a slightly expanded vessel with a smooth contour. Score 4 occurs when a perirectal vessel has an irregular contour or nodular expansion with internal intermediate T2 signal intensity [22]. A score of 0 or 1 is predictive of absence of EMVI at histopathology whereas scores of 3 or 4 are suggestive of the presence EMVI (Fig. 4). A score of 2 was initially defined as a negative [22], but subsequent research has shown that one-third of cases with this score can be positive histpathologically, so a score of 2 is often considered equivocal [26].

Beyond the 5-point grading, additional features of MR detected EMVI have been investigated, including the significance of the number and diameter of involved vessels, the location (e.g., originating from the upper, middle, or lower rectum), and the causes of vascular involvement (i.e., EMVI arising from the primary tumor as opposed to involved lymph nodes or tumor deposits defined as satellite peritumoral nodules of carcinoma in the mesorectal fat remote from the primary tumor, with no sign of residual lymph nodes or identifiable vessels or neural structures [33]). EMVI arising from the upper rectum and larger vessels is associated with a greater risk of poor prognosis or distant metastasis [24, 34]. EMVI arising from lymph nodes or tumor deposits has been shown to carry a prognosis similar to EMVI arising from the primary rectal tumor [34].

**Fig. 5:**
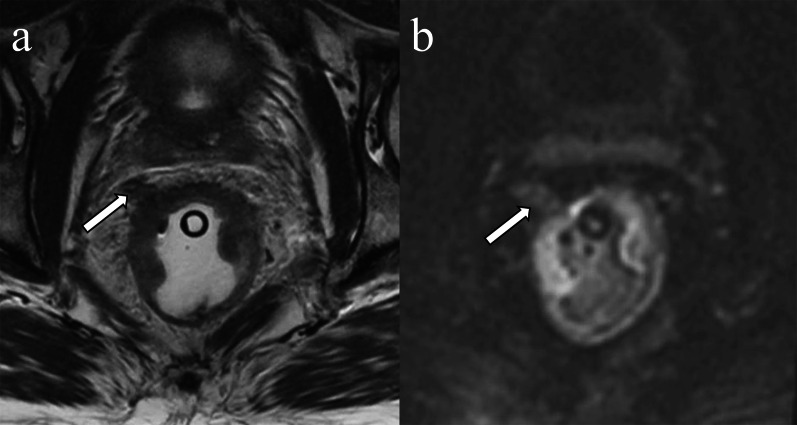
Extramural invasion on diffusion weighted imaging. A 41-year-old man with rectal adenocarcinoma. T2-weighted image demonstrates a distended perirectal vessel with a lack of flow void and central tumor signal intensity which is contiguous with the primary rectal tumor (**a** arrow). Diffusion-weighted image shows a high signal intensity cord-like structure contiguous with the primary rectal tumor (**b** arrow)

Diffusion-weighted images (DWI) often depict the intravenous tumoral component as intermediate or high tumor-signal intensity within normal or slightly expanded extramural vessels adjacent to the primary tumor (Fig. 5). Despite the potential pitfalls of DWI such as susceptibility artifact, limited spatial resolution, T2 shine through, and fibrosis, DWI can be helpful in identifying EMVI at both initial staging and restaging after neoadjuvant therapy [35, 36].

**Fig. 6:**
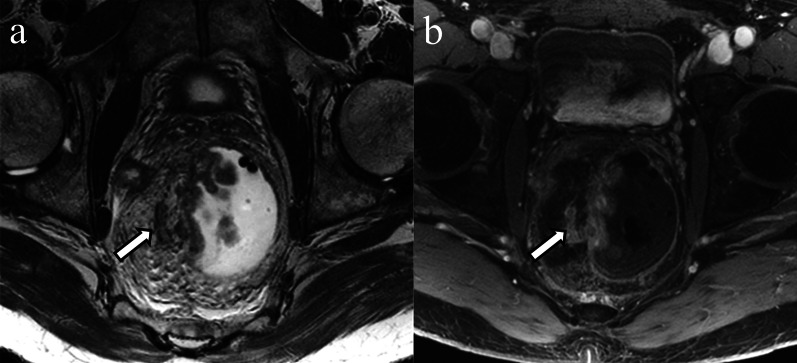
Extramural invasion on contrast-enhanced T1-weighted image. A 56-year-old male with rectal adenocarcinoma. The dilated vessel which is in continuity with the tumor has an irregular margin and contains intermediate signal intensity rather than flow void on the T2-weighted image (**a** arrow). The tumor in the vessel enhances on the contrast-enhanced T1-weighted image (**b** arrow)

On contrast-enhanced T1 weighted images (CE-T1WI), EMVI can be identified as either enhancing tumor within the vessel or as a non- or hypoenhancing intraluminal filling defect [26] within an expanded vessel (≥ 3 mm), that is contiguous with the primary tumor (Table 1) [30]. The delayed phase of contrast enhancement can be helpful as intravenous mixing of contrast during early phases of enhancement in a normal vein may mimic EMVI [41]. CE-T1WI has not been shown to increase accuracy for rectal cancer staging/restaging, and its routine use remains controversial [37–39] as concluded by expert panels from Europe [40] and North America [41]. While 65% of North American panelists reported that they use gadolinium-based contrast media in MRI examinations for rectal cancer, only 29% of European panelists reported doing so [42]. However, CE-T1WI may be a helpful adjunct when the absence/presence of EMVI is equivocal by T2WI [26] (Fig. 6).

**Table 1:** Imaging findings of extramural venous invasion

Pulse sequences or modality	Findings contiguous with primary tumor
T2WI	Intermediate signal intensity with slightly expanded vessels (score 3) or nodular, bead- or worm-shaped structure irregular margin (score 4)
DWI	Intermediate to high signal intensity in the expanded vessels at the site consistent with findings on T2WI
CE-T1WI	Filling defect in the vessel, +/- vessel dilation Tumor signal intensity within the expanded vessel (s)
CT	Heterogeneously enhancing, serpentine, cord-like expanded structure with irregular margin

These findings contiguous with the primary lesion invading beyond the muscularis propria

CE-T1WI, contrast enhanced T1-weighted image; DWI, diffusion-weighted image; T2WI, T2-weighted image

### Performance of rectal cancer MRI to detect EMVI

Brown et al. showed that rectal cancer MRI revealed 83% of histopatologically-detected EMVI with a diameter greater than 3 mm [8]. A recent meta-analysis by Kim et al. showed that MRI had a pooled sensitivity of 61% and specificity of 87% for detecting EMVI in colorectal cancer using histopathology as the reference standard [43]. Table 2 shows sensitivity, specificity, and inter-observer agreement of MR for detection of EMVI in rectal cancer using T2WI alone or T2 combined with DWI or CE-T1WI before and after neoadjuvant therapy [8, 22–32, 44]. Inter-observer agreement for assessing EMVI employing the 5-point scoring system is variable (κ = 0.372–0.828), even when T2WI is used alone [26–32].

**Table 2:** Estimated Performance of MRI for extramural venous invasion

Authors	Year	No. of patients	No. of readers	Pulse sequences	Sensitivity	Specificity	Inter-observer agreement
*Before neoadjuvant therapy*							
Smith [22]	2008	142	1	T2WI	0.62	0.88	NA
Koh [23]	2008	79	NA	T2WI	1.00	0.89	NA
Sohn [24]	2015	447	NA	T2WI	0.282	0.94	NA
Jhaveri [26]	2016	49	2	T2WI, CE-T1WI	T2WI alone: 0.43–0.50 T2WI + CE-T1WI: 0.57	T2WI alone: 0.96–1.00 T2WI + CE-T1WI: 0.96	T2WI alone: 0.828 T2WI + CE-T1WI: 0.858
Liu [27]	2016	183	2	T2WI	0.617	0.820	0.780
Yu [28]	2016	86	2	T2WI	0.583	0.920	0.803
Lee [29]	2018	200	2	T2WI	0.9048	0.4114	0.801
Liu [30]	2016	59	2	T2WI, CE-T1WI	T2WI alone: 0.500–0.722 CE-T1WI alone: 0.556 T2WI + CE-T1WI:0.778–0.833	T2WI alone: 0.732–0.780 CE-T1WI alone: 0.659–0.683 T2WI + CE-T1WI: 0.732–0.756	T2WI alone: 0.603 CE-T1WI alone: 0.216 T2WI + CE-T1WI: 0.413
Ahn [32]	2019	79	2	T2WI, DWI	T2WI alone: 0.80–0.85	T2WI alone: 0.53–0.75	T2WI alone: 0.704
					T2WI + DWI: 0.90	T2WI + DWI: 0.54–0.66	T2WI + DWI: 0.611
Bae [31]	2019	147	3	T2WI, CE-T1WI	T2WI + CE-T1WI: 0.720–0.840	T2WI + CE-T1WI: 0.454–0.856	T2WI + CE-T1WI: 0.376–0.693
Fornell-Perez [45]	2020	54	3	T2WI, DWI	T2WI alone: 0.571 T2WI + DWI: 0.476	T2WI alone: 0.759 T2WI + DWI: 0.872	T2WI alone: 0.483 T2WI + DWI: 0.438
*After neoadjuvant therapy*							
Jhaveri [26]	2016	20	2	T2WI, CE-T1WI	T2WI alone: 0.29	T2WI alone: 0.95–1.00	T2WI alone: 0.781
					T2WI + CE-T1WI: 0.43–0.57	T2WI + CE-T1WI: 0.95	T2WI + CE-T1WI: 0.604
Lee [29]	2018	200	2	T2WI	0.7619	0.7975	0.736
Fornell-Perez [45]	2020	46	3	T2WI, DWI	T2WI alone: 0.667 T2WI + DWI: 0.778	T2WI alone: 0.829 T2WI + DWI: 0.915	T2WI alone: 0.372 T2WI + DWI: 0.361
Bae [31]	2019	75	3	T2WI, CE-T1WI	T2WI + CE-T1WI: 0.667–0.792	T2WI + CE-T1WI: 0.490–0.922	T2WI + CE-T1WI: 0.383–0.693

CE-T1WI, contrast enhanced T1-weighted image; DWI, diffusion-weighted image; T2WI, T2-weighted image

Data regarding the value of DWI in detecting EMVI are conflicting. Ahn et al. reported that the addition of DWI reduced inter-observer agreement with no additional diagnostic benefit [32]. Conversely, Fornell-Perez et al. reported that the addition of DWI improved diagnostic accuracy in detecting EMVI, especially in post chemoradiation therapy patients [45]. Interestingly, Coruh et al. found that the ADC (apparent diffusion coefficient) values of the primary tumor are significantly lower in EMVI positive tumors [46].

Jhaveri et al. showed that the sensitivity and specificity of rectal MRI for EMVI did not significantly change with the addition of contrast-enhancement (for initial or restaging studies), but that interobserver agreement remained good [26]. Similarly the meta-analysis by Kim et al. showed no significant improvement in performance with gadolinium contrast or DWI [43]. In contrast, Lui et al. showed that contrast enhancement may improve sensitivity but at the cost of reduced interobserver agreement [30].

### EMVI in computed tomography

**Fig. 7:**
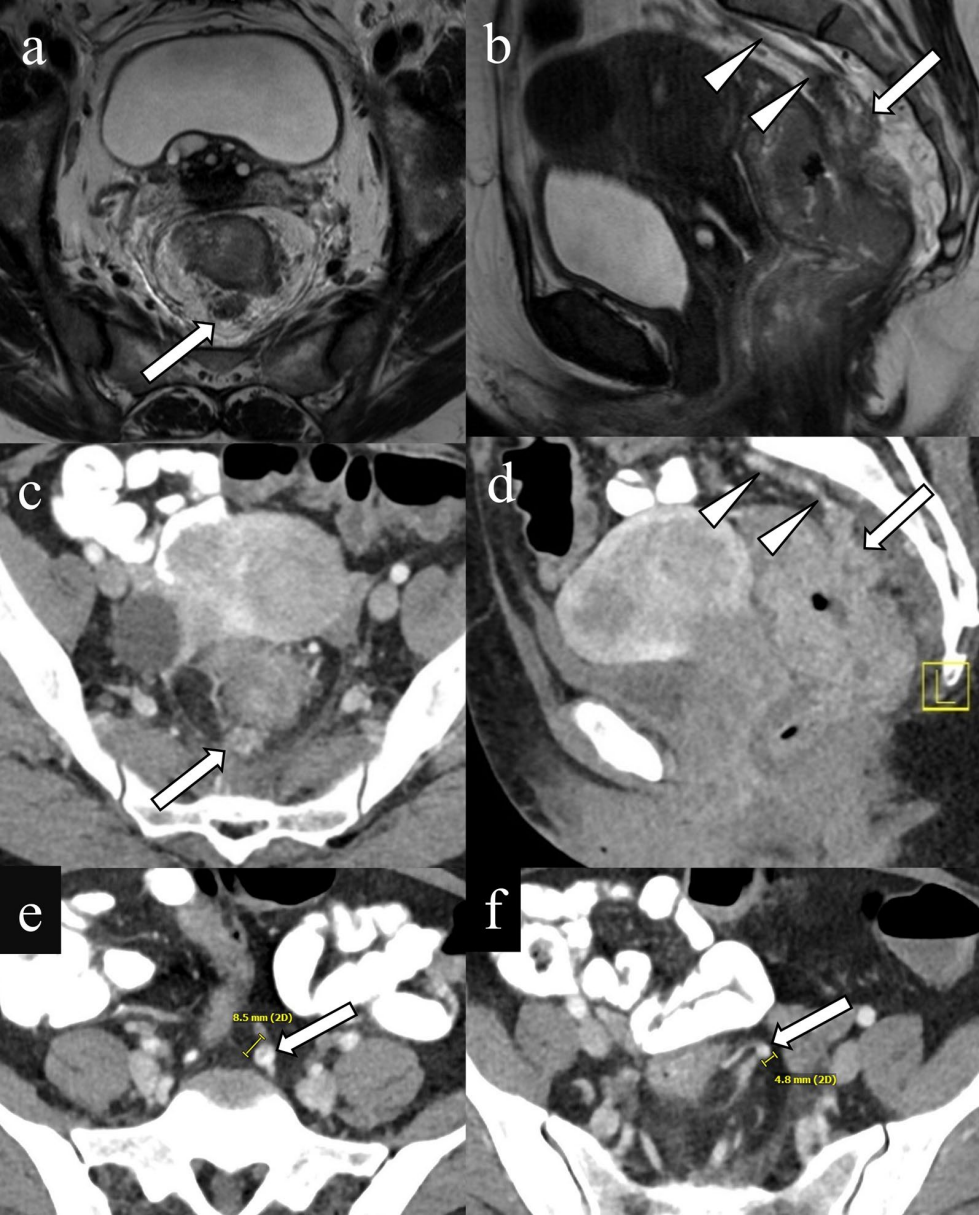
Extramural venous invasion on CT. A 47-year-old female with rectal adenocarcinoma. A nodule with an irregular margin containing intermediate signal intensity is observed in the mesorectum on axial T2WI (**a** arrow). Sagittal T2WI reveals a cord-like structure with tumor signal intensity in the superior rectal vein (**b** arrow) which drains to the inferior mesenteric veins (**b** arrowheads) Similarly, contrast-enhanced CT demonstrates an enhancing irregular nodule within the posterior mesorectum on the axial image (**c** arrow). On the sagittal image a cord-like nodular mass is contiguous with the dilated superior rectal vein (**d** arrow) and inferior mesenteric veins (**d** arrow heads) on the sagittal image. The diameters of the inferior mesenteric and superior rectal veins are 8.5 mm (**e** arrow) and 4.8 mm (**f** arrow), respectively, which is suggestive of the presence of EMVI

Compared with MRI, the role of CT in assessing EMVI is limited because of its lower contrast resolution. However, CT can be an alternative to assess EMVI in patients who have a contraindication to MRI or who are unable to undergo MRI due to claustrophobia. On CT, EMVI is often seen as a heterogeneously enhancing, serpentine cord-like structure connecting veins with the irregular, contiguous margins of the primary tumor (Fig. 7). Ortega et al. investigated the diagnostic accuracy of CT-detected EMVI in rectal cancer using venous distension and intravascular tumor enhancement as the imaging criteria and using MRI-detected EMVI as the reference standard. They reported a high specificity of 100%, but low sensitivity of 14% and NPV of 47% [47]. Routine mention of CT-detected EMVI in clinical reports is not required; however, the presence of CT-detected EMVI can aid in therapeutic decision making in patients who have a contraindication to MRI, so reporting EMVI may be helpful when detected. Further investigation is warranted to determine the clinical significance of CT-detected EMVI.

Dilation of the superior rectal and inferior mesenteric veins may help to predict EMVI (Fig. 7). Wu et al. reported that using a cut off value of 3.7 mm for the diameter of the superior hemorrhoidal vein, lymphovascular invasion by rectal cancer could be predicted on CT [48]. Similarly, in a study by Coruh et al., the diameter of the superior rectal and the inferior mesenteric veins were significantly larger in patients with EMVI on CT. Cutoff values of 3.95 mm for the superior rectal vein and 5.95 mm for the inferior mesenteric vein predicted EMVI with 93.3% and 93.3% sensitivity and 67.9% and 71.4% specificity, respectively [46]. It was hypothesized that the presence of an intravenous tumor may result in increased blood flow in the major drainage pathways of the rectum such as the superior rectal and inferior mesenteric veins, and that dilation may be an indirect indicator of EMVI. Likewise, increased venous drainage caused by tumor neoangiogenesis may result in vein dilation.

### Clinical significance of MRI-detected EMVI

Multiple studies have demonstrated the clinical significance of EMVI detected on MRI as a predictor of poor prognosis or biologic aggressiveness in rectal cancer. MRI-detected EMVI is generally present in patients with T3 and nodal disease and is consequently, at least in North America, used to determine which patients will benefit from neoadjuvant therapy. Conversely, the overuse of neoadjuvant therapy in rectal cancer patients does not improve survival and can result in bowel and sexual dysfunction [49], and a recent European clinical trial has advocated that rectal cancer MRI demonstrating absence of EMVI may facilitate patient selection for primary surgery [50].

### Predicting survival

**Fig. 8:**
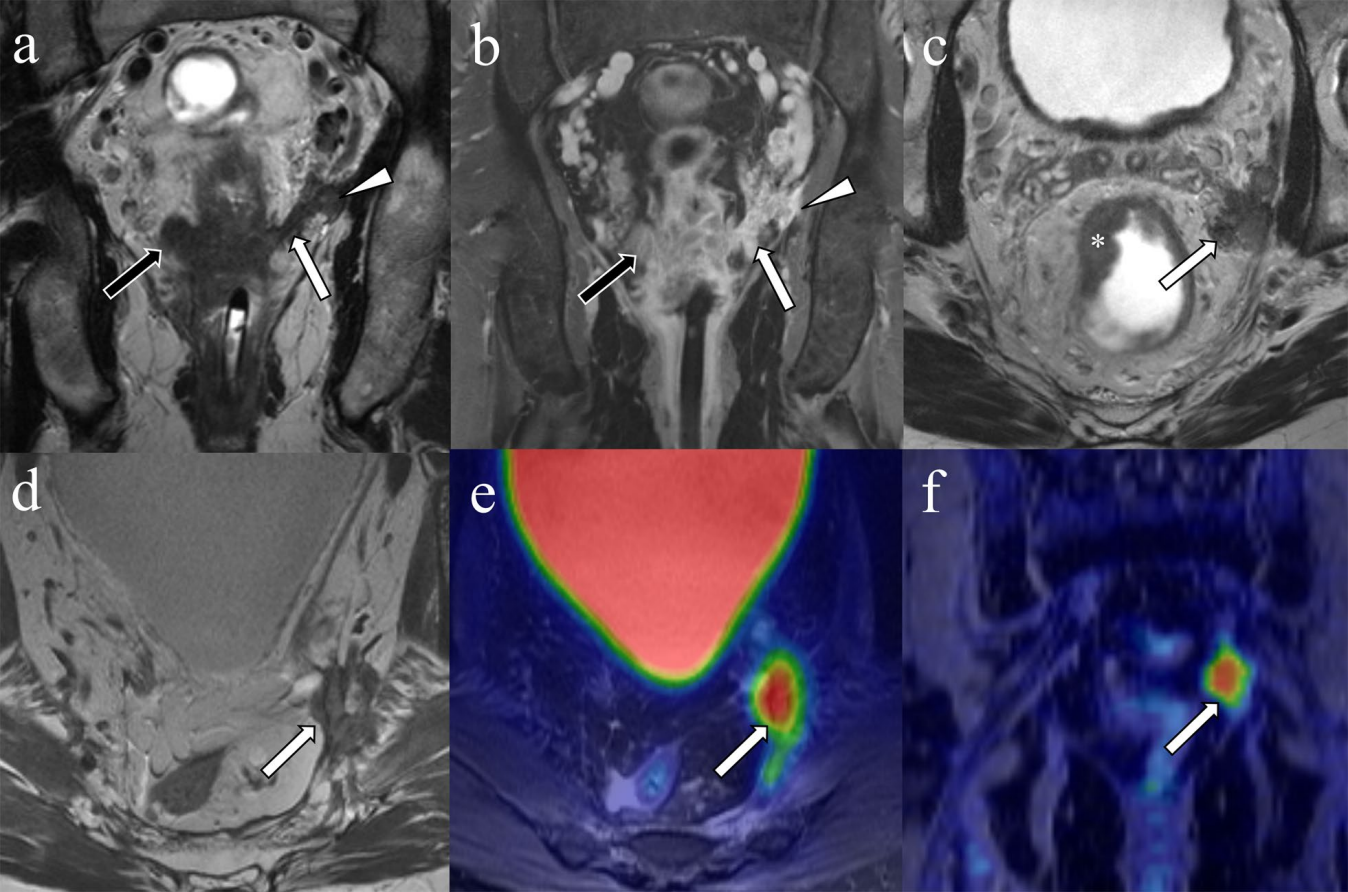
Local recurrence after surgery from residual disease associated with positive circumferential margin due to extramural venous invasion. A 58-year-old man with rectal cancer. T2-weighted (**a**) and postcontrast (**b**) coronal image before treatment demonstrate nodular-shaped structure (black arrows) on the right extending to the mesorectal fat tissue, and worm-shaped (white arrows) structure on the left with tumor signal intensity arising from the primary lesion, indicating extramural venous invasion (EMVI) extending the mesorectal fascia (arrow heads). Axial T2-weighted image shows a primary tumor (**c** asterisk) and an irregular tumoral deposit in and abutting the mesorectal facia near the left pelvic sidewall (**c** arrow). Axial and coronal ^18^F-FDG-PET/MRI images 23 months after surgery following neoadjuvant therapy show FDG avidity corresponding to a developed nodular recurrence at the same location (**d**–**f** arrows)

MRI-detected EMVI is reported to be an independent significant prognostic factor for overall disease-free survival and systemic recurrence in rectal cancer [51]. In locally advanced rectal cancer, MRI-detected EMVI predicted decreased disease-free survival (hazard ratio: 2.46) [52]. In another study, MRI-detected EMVI before neoadjuvant therapy was an independent poor prognostic factor for progression-free survival (hazard ratio: 1.85) [53], disease-free survival (hazard ratio: 1.35–31.33) [29, 54–58] and overall survival (hazard ratio: 1.18–2.90) (Fig. 8) [29, 59]. MRI-detected EMVI after neoadjuvant chemotherapy has also been shown to be a predictor of decreased disease-free survival (hazard ratio: 1.97–2.68) [25, 29], recurrence-free survival (hazard ratio: 2.74) [60] and overall survival (hazard ratio: 1.98–4.23) [29, 59, 60] (Table 3).

**Table 3:** Relationship between MRI-detected extramural venous invasion  and patient outcome

Author	Year	No. of patients	Neoadjuvant therapy	Endpoint	Hazard ratio (95% CI)
*EMVI before neoadjuvant therapy*					
Chand [54]	2014	478	CRT (69.2%: 331/478)	DSF (3 year)	2.08 (1.10–3.07)
Sclafani [53]	2014	269	CRT	PFS (5 year)	1.85 (1.12–3.05)
Jalil [59]	2016	56	CRT	OS	2.90 (1.00–8.37)
Patel [55]	2017	46	Chemotherapy	DSF (3 year)	31.33 (2.31–425.4)
Lee [29]	2018	200	CRT	DSF (3 year)	1.35 (0.64–2.82)
				OS	1.18 (0.51–2.77)
Jia [52]	2018	185	No	DSF (3 year)	2.46 (1.28–4.74)
Meng [56]	2019	115	CRT	DSF (3 year)	2.50 (1.24–5.01)
Gu [57]	2019	146	short course CRT	DSF (3 year)	3.56 (2.03–13.32)
Meng [58]	2019	171	CRT	DSF (3 year)	2.59 (1.40–4.80)
*EMVI after neoadjuvant therapy*					
Chand [25]	2015	188	CRT	DSF (3 year)	1.97 (1.01–3.90)
Jalil [59]	2016	56	CRT	OS	4.23 (1.41–12.69)
Lee [29]	2018	200	CRT	DSF (3 year)	2.68 (1.37–5.27)
				OS	1.98 (0.88–4.42)
Shiraishi [60]	2019	102	Chemotherapy	RFS (5 year)	2.74 (1.36–5.50)
				OS	3.15 (0.91–10.89)

CRT, chemoradiation therapy; DSF, disease-free survival; OS, overall survival; PFS, progression-free survival; RFS, recurrence-free survival

Rectal cancer MRI can also demonstrate in situ evidence of response or progression of EMVI after neoadjuvant therapy. Also, a change in MRI-detected EMVI status from positive to negative has been shown to predict histopathologic response [61]. Additionally, patients who have significant response of MRI-detected EMVI after neoadjuvant therapy, defined as more than 50% of the intravascular tumor content converted to low signal intensity fibrosis (i.e., signal intensity as low as the muscularis propria), have improved disease-free survival [62]. Regarding surgery, EMVI at initial staging MRI was a risk factor of failure to convert from positive to negative circumferential resection margin by neoadjuvant chemoradiotherapy [63]. Even when considering patients with positive resection margins, patients with EMVI have decreased survival compared to those without EMVI [64].

### Predicting lymph node and distant metastases

In one study, EMVI score correlated with histologic lymph node stage [28]. In other studies, MRI-detected EMVI was found to be present in about a quarter of patients with rectal cancer, and had a specificity of about 81% for predicting N2 [23] and 88% for regional lymph node metastases [23, 27]. MRI detected-EMVI is also associated with a significantly higher risk for both synchronous [24, 65] and metachronous metastasis [66]. Approximately 25% of rectal cancer patients with EMVI on MRI developed subsequent liver and lung metastases at 1-year, compared to about 7% of patients without EMVI (Relative risk: 3.70) [66]. These findings are in keeping with the concept that EMVI may be the first step in hematogenous metastasis [17]. Table 4 summarizes the results of the studies correlating MRI-detected EMVI with lymph node and distant metastases.

**Table 4:** Risk of lymph node and distant metastasis with MRI-detected extramural venous invasion

Authors	Year	No. of patients	Treatment	MRI timing	Endopoint	Summary of results
Koh [23]	2008	79	No	Pre surgery	Nodal disease	Sv: 0.56 (0.79–0.96) Sp: 0.81 (0.69–0.90)
Liu [27]	2016	183	No	Pre surgery	Nodal disease	OR: 4.22 (1.79–9.95)
Bugg [66]	2014	202	No data	Pre surgery	Metachronous metastasis	RR: 3.70 (95%CI: NA)
Sohn [24]	2015	447	nCRT (9.2%: 41/447)	Pre nCRT	Synchronous metastasis	OR:3.02 (1.65–5.51)

nCRT, neoadjuvant chemoradiation therapy; R, odds ratio; RR, relative risk; Sp, specificity; Sv, sensitivity

### Extramural venous invasion of other rectal tumors

**Fig. 9:**
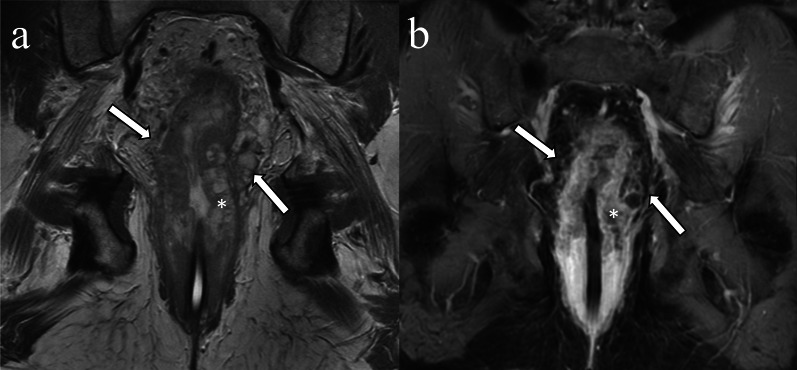
Extramural venous invasion in mucinous adenocarcinoma. A 53-year-old man with rectal mucinous adenocarcinoma. The circumferential rectal tumor shows high signal intensity on coronal T2WI (**a** asterisk). Peripheral heterogeneous enhancement is observed on contrast enhanced T1WI (**b** asterisk). The intravenous component demonstrates signal intensity and enhancement similar to the primary lesion (**a**, **b** arrows)

**Fig. 10:**
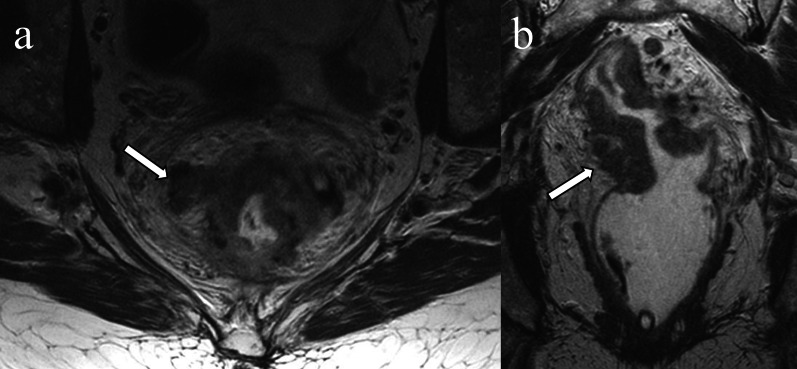
Extramural invasion in squamous cell carcinoma. A 58-year-old male with rectal squamous cell carcinoma. A nodular, elongated structure extends cranially from the right side of the circumferential rectal tumor (**a**, **b** arrow)

**Fig. 11:**
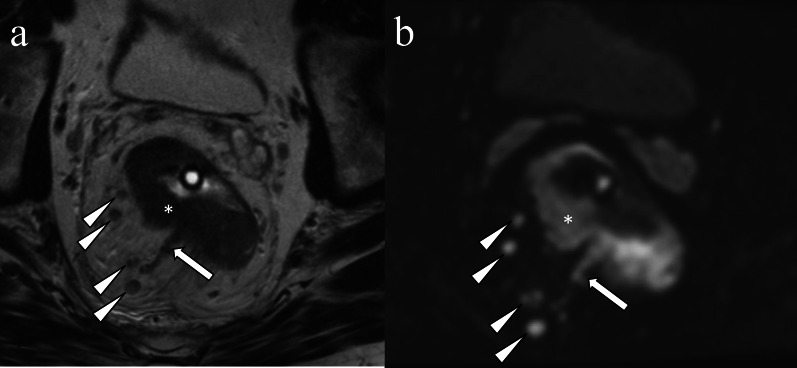
Extramural venous invasion in neuroendocrine tumor. A 50-year-old man with poorly differentiated neuroendocrine tumor, large-cell type. On the T2WI a tubular structure with irregular margins and signal intensity similar to the tumor (**a** arrow) extends from the rectal mass (**a** asterisk). The primary lesion (**b** asterisk) and extramural venous invasion (**b** arrow) are more conspicuous on DWI. Mesorectal lymph nodes which were histopathologically proven to be nodal metastases are depicted on T2WI and DWI (**a**, **b** arrowheads)

Rectal neoplasms other than usual adenocarcinomas such as squamous cell carcinoma, mucinous adenocarcinoma, and neuroendocrine tumor also can invade perirectal vessels (Figs. 9, 10, 11).

Mucinous rectal carcinoma is a distinct pathologic subtype of rectal cancer defined as a tumor composed of greater than 50% extracellular mucin and neoplastic epithelium surrounded by extracellular mucin lakes on histopathology [13]. The primary tumor is markedly hyperintense on T2WI owing to extracellular mucin [67]. Following administration of IV contrast material, the mucinous tumor has peripheral and heterogeneous enhancement. Mucinous rectal adenocarcinomas commonly have lower signal on higher b value DWI and a higher mean apparent diffusion coefficient compared to non-mucinous rectal cancers [68]. When EMVI is present, the intravenous tumor component shows the same signal characteristics as the primary tumor (Fig. 9). MRI is considered superior to biopsy in identifying mucinous malignancy secondary to sampling errors with biopsy [69]. Metachronous metastases are seen more often in mucinous carcinoma than non-mucinous carcinoma regardless of whether EMVI is present or absent [70].

Rectal squamous cell carcinoma is a rare tumor, which accounts for less than 1% of colorectal malignancies [71]. Risk factors include chronic infection, smoking, human immunodeficiency virus, and human papillomavirus [72]. Owing to its low prevalence, the imaging characteristics of squamous cell carcinoma are not well-documented, and the frequency and clinical significance of squamous cell carcinoma-associated EMVI are unknown (Fig. 10).

The rectum is a common site of neuroendocrine neoplasms. Rectal neuroendocrine tumor usually appears as a submucosal nodule or focal area of plaque-like wall-thickening; however, less commonly it can present as a large ulcerating, avidly enhancing, invasive mass [73]. The incidence of rectal neuroendocrine tumor has been increasing due to incidental detection, especially for small neoplasms. Large rectal neuroendocrine tumors can be difficult to differentiate from adenocarcinoma. EMVI of rectal neuroendocrine tumors in cross-sectional imaging has also not been well-described (Fig. 11). Neuroendocrine neoplasms smaller than 1.0 cm can be treated with resection; however, lymphatic and venous invasion are predictors of metastasis [74], and salvage surgery is recommended in patients with lymphovascular invasion [75].

## Conclusion

MRI-detected EMVI correlates closely with histopathological EMVI and is a predictor of lymph node and distant metastases, tumor recurrence, and poor prognosis. Therefore, it is important to evaluate for the presence of EMVI on rectal cancer MRI examinations before and after neoadjuvant therapy to determine risk-stratification and therapeutic options. Despite the clinical significance of MRI-detected EMVI, inter-observer variability in assessing its presence or absence is problematic both at initial staging and after neoadjuvant treatment. Radiologists should therefore be familiar with the imaging features of EMVI and its implications for patient management. Findings of EMVI include expanded vessel caliber adjacent to the primary tumor, intermediate tumor signal intensity in the vessel, and irregular vessel margin. DWI and contrast enhanced T1 weighted images may be helpful adjuncts to T2WI and may help improve reader confidence in select cases. Further investigation is necessary to determine if multi-parametric MRI improves diagnostic performance without compromising interobserver agreement. In addition, further investigation is needed to assess the clinical importance of CT-detected EMVI and to determine whether detailed features of EMVI, including location, vessel diameter, and the number of involved vessels can improve risk-stratification.

## Abbreviations

CE-T1WI: Contrast enhanced T1-weighted image
CT: Computed tomography
DWI: Diffusion-weighted image
EMVI: Extramural venous invasion
MERCURY: Magnetic resonance imaging and rectal cancer European equivalence
MRI: Magnetic resonance imaging
T2WI: T2-weighted image
